# Luteolin and neuroinflammation: a multi-target therapeutic strategy for central nervous system disorders

**DOI:** 10.3389/fphar.2026.1844585

**Published:** 2026-05-26

**Authors:** Jianting Huang, Miaomiao Yu, Yanan Zou, Lezhen Wang, Yuan Ma, Yue Ma, Xiangyi Kong, Fei Rong

**Affiliations:** 1 Department of Anesthesiology, Qilu Hospital (Qingdao), Cheeloo College of Medicine, Shandong University, Qingdao, China; 2 Radiology Department, Qingdao Central Hospital, University of Health and Rehabilitation Sciences, Qingdao, China; 3 Qingdao Mental Health Center, Qingdao, China

**Keywords:** blood–brain barrier, luteolin, neuroinflammation, neurological disorders, reactive oxygen species

## Abstract

Neuroinflammation is a fundamental pathological hallmark driving the initiation and progression of various neurological disorders. Luteolin, a natural flavonoid abundant in medicinal plants, fruits, and vegetables, exerts multifaceted neuroprotective effects across diverse experimental disease models. Its beneficial activities are mediated through complementary mechanisms, including suppression of aberrant microglial activation, modulation of pro-inflammatory signaling pathways, and enhancement of endogenous antioxidant defenses. These integrated actions attenuate inflammatory mediator release, reduce oxidative stress-induced neuronal damage, and inhibit apoptosis, thereby counteracting neuroinflammation-driven pathology. This review synthesizes current knowledge on luteolin’s protective roles in central nervous system (CNS) disorders. It elucidates underlying molecular mechanisms, encompassing regulation of key signaling cascades such as NF-κB, MAPK, and Nrf2, as well as its impact on cellular processes including autophagy and mitochondrial function. Critical challenges hindering clinical translation—notably limited oral bioavailability and restricted blood-brain barrier (BBB) permeability—are systematically discussed to guide future research. A comprehensive understanding of luteolin’s pleiotropic pharmacological actions will not only enhance knowledge of its therapeutic potential but may also facilitate the development of novel preventive and therapeutic strategies for neuroinflammatory and neurodegenerative diseases.

## 1 Introduction

Neuroinflammation is characterized by a transition from an initial acute response to a prolonged and dysregulated activation state of resident immune cells within the CNS, primarily microglia and astrocytes (Leng and Edison, 2021; Shi and Yong, 2025). It constitutes a pivotal element in the pathogenesis and progression of numerous neurological disorders and has emerged as a core pathological feature of conditions such as Alzheimer’s disease (AD), Parkinson’s disease (PD), amyotrophic lateral sclerosis (ALS), and ischemic stroke. Unlike acute neuroinflammation, which is self-limiting and primarily serves pathogen elimination and tissue repair, chronic neuroinflammation involves a sustained, uncontrolled immune response that fosters a neurotoxic microenvironment (Kong et al., 2024b; Chagas et al., 2025; Lonkar et al., 2025; Hoffman et al., 2026). During this process, aberrantly activated microglia and astrocytes continuously release elevated levels of pro-inflammatory cytokines including TNF-α, IL-1β, IL-6, chemokines, and reactive oxygen/nitrogen species (ROS/RNS) (Geng et al., 2025; Kakkar et al., 2025). These mediators directly inflict neuronal damage, disrupt synaptic structure and function, inhibit neurogenesis, and ultimately accelerate neurodegenerative cascades. Importantly, neuroinflammation is not merely a consequence of neural injury but an active contributor to disease progression (Kong et al., 2024a; Tastan and Heneka, 2024). It frequently engages in a vicious cycle with other pathological hallmarks—including aberrant protein aggregation (e.g., Aβ, α-synuclein), mitochondrial dysfunction, oxidative stress, and BBB disruption—collectively exacerbating disease deterioration (Calderone et al., 2024; Pluta, 2025). Current clinical intervention strategies targeting neuroinflammation remain limited. Conventional anti-inflammatory drugs, including glucocorticoids and non-steroidal anti-inflammatory drugs (NSAIDs), are hampered by poor penetration of the intact BBB, narrow therapeutic windows, and significant systemic side effects associated with long-term use, thereby restricting their application in chronic neurodegenerative conditions (Chen et al., 2023; Mallick et al., 2025). Consequently, the development of novel anti-neuroinflammatory agents endowed with favorable BBB permeability, multi-target regulatory capabilities, and a high safety profile has become a critical research priority in contemporary neuropharmacology (Kim and Lee, 2024; Giang et al., 2025).

**FIGURE 1:**
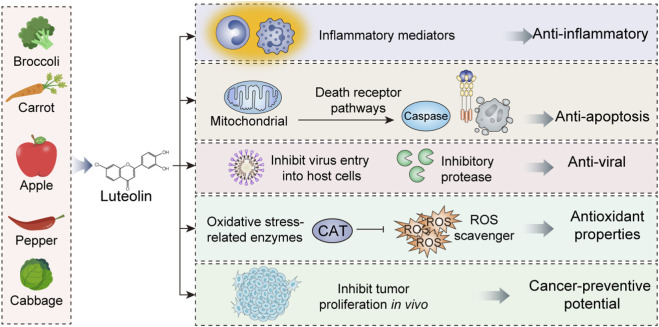
Multifaceted Biological Activities of Luteolin and Their Potential Implications in Neurological Disorders. Luteolin, a natural flavonoid found in carrots, broccoli, peppers, cabbage, and apples, exhibits four major pharmacological activities relevant to neurological disorders: 1 Anti-inflammatory–suppresses pro-inflammatory mediators, alleviating neuroinflammation; 2 Anticancer–inhibits tumor cell proliferation, potentially contributing to chemoprevention of CNS tumors; 3 Antioxidant–scavenges ROS and modulates oxidative stress-related enzymes (e.g., catalase), reducing neuronal oxidative damage; 4 Anti-apoptotic & antiviral–blocks mitochondrial and death receptor-mediated apoptotic pathways, protecting neurons from cell death. Additionally, it inhibits viral entry into host cells and viral protease activity, which may have implications for viral encephalopathy, HIV, and other neurotropic viral infections.

In this context, natural bioactive molecules have garnered considerable attention due to their pleiotropic effects, low toxicity, and favorable biocompatibility. Luteolin (3′,4′,5,7-tetrahydroxyflavone), a dietary flavonoid abundantly present in vegetables, fruits, and various medicinal plants, exhibits significant anti-inflammatory, antioxidant, and neuroprotective activities (Jayawickreme et al., 2024; Zhu et al., 2024). Its relatively low molecular weight and moderate lipophilicity confer a notable capacity to penetrate the BBB, enabling it to reach effective concentrations within the CNS. Preclinical studies consistently demonstrate that luteolin potently inhibits the overactivation of microglia and astrocytes (Zhou et al., 2021; Kou et al., 2022). Its mechanisms involve the modulation of multiple key inflammatory signaling pathways, including the suppression of nuclear factor-kappa B (NF-κB) and NOD-like receptor protein 3 (NLRP3) inflammasome activation, downregulation of mitogen-activated protein kinase (MAPK) signaling, and enhancement of the nuclear factor erythroid 2-related factor 2 (Nrf2)-mediated antioxidant defense system (Qiao et al., 2023; Bottcher et al., 2025; Gu and Pang, 2025). Through these multi-target effects, luteolin effectively reduces the production of pro-inflammatory cytokines and ROS, mitigates neuroinflammation-mediated synaptic loss and neuronal death, and exhibits clear neuroprotective effects in various animal models of AD, PD, and stroke (Goyal et al., 2024; Younis et al., 2024; Singh et al., 2025). Luteolin has also been reported to inhibit viral entry and replication, including neurotropic viruses such as Japanese encephalitis virus. These antiviral properties may have implications for viral encephalitis and other infection-related neurological conditions (Fan et al., 2016; Peng et al., 2017; Men et al., 2023). Although existing evidence amply demonstrates the potential of luteolin in regulating neuroinflammation, several critical issues remain to be systematically addressed. First, the mechanisms of luteolin across different neuroinflammatory disease models share commonalities yet exhibit disparities, and an integrated, cross-disease analysis of its signaling network is lacking. Second, its pharmacokinetic properties, such as oral bioavailability, tissue distribution, and metabolic pathways, require further optimization to enhance its *in vivo* efficacy (Yis et al., 2018; Taheri et al., 2021). Third, there is a paucity of high-quality clinical evidence to support its translation into clinical application (Ali and Siddique, 2019; Jayawickreme et al., 2024) (Figure 1).

This review aims to systematically synthesize recent research findings, comprehensively elucidate the molecular mechanisms underlying luteolin’s anti-neuroinflammatory effects, and critically evaluate its therapeutic potential in the specific contexts of AD, PD, stroke, and multiple sclerosis (MS). Furthermore, this article will delve into the latest advancements in improving its bioavailability and targeting through formulation strategies (e.g., nano-delivery systems, structural modification), analyze the major challenges confronting its clinical translation, and propose future research directions. The overarching goal is to provide a theoretical basis and strategic reference to facilitate the progression of this natural compound towards clinical therapy for neuroinflammatory disorders.

## 2 Luteolin suppresses pro-inflammatory activation of CNS immune cells

### 2.1 Macrophages

Macrophages serve as functionally versatile sentinels of the innate immune system, playing critical roles in maintaining tissue homeostasis, regulating inflammatory responses, and orchestrating tissue repair (Cheng et al., 2021; Bernier et al., 2025). Classically activated (M1) macrophages, typically induced by stimuli such as lipopolysaccharide (LPS) and interferon-γ (IFN-γ), are potent producers of pro-inflammatory cytokines (e.g., TNF-α, IL-1β, IL-6), as well as NO and ROS, which are essential for host defense (Leng and Edison, 2021; Dahdah et al., 2024; Behmoaras et al., 2025). In contrast, alternatively activated (M2) macrophages, induced by IL-4 or IL-13, promote the resolution of inflammation, tissue remodeling, and immune regulation through the secretion of factors such as IL-10 and TGF-β (Isik et al., 2023; Sun and Jiang, 2024; Butovsky et al., 2026). Flavonoids, a large group of dietary polyphenols, have garnered significant attention for their broad spectrum of biological activities, including potent anti-inflammatory and immunomodulatory effects. Among these, luteolin stands out as a powerful modulator of macrophage-mediated inflammation. Research indicates that luteolin inhibits CD74 expression and disrupts the assembly of the CEBPB-p65 complex, thereby preventing p65 nuclear translocation and subsequent activation of the NF-κB signaling cascade in macrophages (Peng et al., 2026). This action on CD74^+^ macrophages plays a significant role in treating synovial inflammation in osteoarthritis (Peng et al., 2026). In macrophages stimulated with LPS plus IFN-γ to induce M1 polarization, or with IL-4 to induce M2 polarization, luteolin treatment markedly decreases the expression of M1-type pro-inflammatory mediators and the surface marker CD86, while significantly increasing the expression of M2-associated anti-inflammatory factors and CD206. Furthermore, it downregulates p-STAT3 and upregulates p-STAT6, thereby curbing inflammatory progression (Wang et al., 2020). In colitis, luteolin inhibits the phosphorylation of IKKα/β, IκBα, and p65, while simultaneously preventing IκBα degradation in LPS-treated RAW264.7 cells and peritoneal macrophages. By antagonizing IKKα/β, it inhibits IKKα/β phosphorylation and subsequent NF-κB activation, thereby preventing macrophage activation and migration. This action alleviates colitis symptoms, restores intestinal barrier integrity, and suppresses pro-inflammatory cytokine production in colon tissue, effectively treating colitis (Xue et al., 2023). Similarly, in ulcerative colitis, luteolin induces a phenotypic switch of macrophages from the M1 to the M2 type by activating the AMPK-PPARγ signaling pathway, effectively mitigating disease progression in a murine model (Yang et al., 2025). In colon cancer, luteolin promotes ferroptosis in tumor cells by facilitating the accumulation of ROS/Fe^2+^, lipid peroxidation, elevated MDA, GSH depletion, and downregulation of GPX4. This synergistically fosters intratumoral M1 macrophage polarization and CD8^+^ T lymphocyte activation, thereby inhibiting colon cancer cell proliferation (Cao et al., 2025). In acute pneumonia induced by *Pseudomonas aeruginosa*, luteolin suppresses the inflammatory response and M1 macrophage polarization by inhibiting the phosphorylation of EGFR, PI3K, AKT, IκBα, NF-κB p65, ERK, c-Jun, and c-Fos in lung tissue. This leads to reduced pulmonary permeability, neutrophil infiltration, production of pro-inflammatory cytokines (IL-1β, IL-6, TNF, and MIP-2), and bacterial burden in lung tissue, effectively alleviating acute lung injury (Gu and Pang, 2025). In periodontal disease, luteolin treatment enhances the expression of CD206 and Arg1, inhibits JAK2/STAT3 phosphorylation, and promotes M2 macrophage polarization. It also reduces ROS, restores mitochondrial membrane potential, decreases mitochondrial fission, and promotes mitochondrial fusion, thereby improving mitochondrial function. These combined effects exert an anti-inflammatory action and suppress the progression of periodontitis (Ma S. et al., 2025). In brief, macrophages serve as key mediators of luteolin’s regulatory effects on neuroinflammation. Anti-inflammatory strategies targeting macrophages, particularly through intervention with bioactive substances like luteolin, offer a potential therapeutic avenue for mitigating neuroinflammation and related systemic inflammation.

### 2.2 Microglia

Microglia, the resident macrophages of the CNS, act as steadfast guardians of neural tissue. Under normal physiological conditions, these highly dynamic cells continuously surveil the neural parenchyma, participating in synaptic pruning, neurogenesis, and the clearance of cellular debris (Chen et al., 2025; Fumagalli et al., 2025; Malcangio and Sideris-Lampretsas, 2025). However, upon encountering pathological stimuli such as infection, trauma, or protein aggregation, microglia rapidly activate and undergo phenotypic transformation accompanied by morphological changes, proliferation, and the release of various signaling molecules. Luteolin, a dietary flavonoid found in celery, peppers, and chamomile, has been demonstrated to be an effective modulator of microglia-mediated neuroinflammation (Dirscherl et al., 2010). Studies show that in LPS-induced cognitive impairment, luteolin exerts a protective effect against neuronal damage by suppressing the levels of inflammatory cytokines such as TNF-α and IL-1β, as well as inflammatory mediators like NO, thereby curbing the overproduction of pro-inflammatory cytokines in the mouse hippocampus and cortex (Zhou et al., 2021). In depression, luteolin treatment interacts with PPARγ to induce a phenotypic shift in pro-inflammatory microglia towards an anti-inflammatory Arg1-expressing phenotype. This alleviates microglial pro-inflammatory responses and reverses phagocytosis-mediated synaptic loss, significantly improving depressive-like behaviors and reducing hippocampal inflammation (Yuan et al., 2025). It has been reported that luteolin treatment reverses symptoms such as weight loss, motor retardation, multi-organ damage, and cognitive deficits in cadmium-exposed mice. The mechanism involves inhibiting the Notch1/Hes1 inflammatory signaling axis while restoring the BDNF-TrkB/AKT1 signaling axis, thereby suppressing pro-inflammatory factor production and neuroinflammation in the hippocampus and prefrontal cortex, ultimately mitigating neurotoxicity (Ma H. Y. et al., 2025). In a zebrafish model of ultraviolet radiation exposure, luteolin activates VEGF and modulates pro-inflammatory cytokine (IL-1β, TNF-α) levels, upregulating the local neuroendocrine axis, blocking apoptosis and lipid peroxidation, downregulating pro-inflammatory cytokine protein expression, and reducing VEGF activation, thus protecting adult zebrafish (Sudhakaran et al., 2023). In PD, luteolin treatment promotes functional recovery and mitigates the loss of dopaminergic neurons. It significantly downregulates TLR4 mRNA and protein expression, inhibits phosphorylation of the NF-κB p65 subunit, and shifts microglial M1/M2 polarization towards the anti-inflammatory M2 phenotype both *in vitro* and *in vivo* (Xie et al., 2024). In a diabetic rat model, luteolin/ZnO-synthesized nanomaterials upregulate miR-124, decrease C/EBPA mRNA, increase Bcl-2, and inhibit apoptosis. Additionally, the luteolin/ZnO composite reduces lipid peroxidation, enhances antioxidant enzyme activity, and diminishes inflammation under oxidative stress (Moustafa et al., 2023). In the treatment of AD, luteolin improves cognitive function in AD model mice by inhibiting CD68 levels, reducing IL-1β production, suppressing ER stress, inhibiting LPS-induced IL-1β expression, preventing ER stress, and curbing microglial activation in the brain (Tana and Nakagawa, 2022). Similarly, combined treatment with luteolin and exercise reverses the increase in Aβ content, the activation of astrocytes and microglia, and the decline in autophagy levels in the hippocampus and cortex induced by Aβ_1_–_42_ oligomers in an AD model, reducing neuroinflammation and thereby slowing the progression of memory dysfunction in AD (Tao et al., 2023). CDKL5 deficiency disorder is a rare and severe neurodevelopmental condition. Luteolin treatment promotes hippocampal neurogenesis, improves dendritic spine maturation and the dendritic arborization of hippocampal and cortical neurons. The inhibition of neuroinflammation ameliorates motor stereotypies, hyperactivity, and memory abilities in *Cdkl5^+/−^* mice (Tassinari et al., 2022). In subarachnoid hemorrhage, luteolin significantly enhances the expression of Nrf2 while concurrently downregulating NLRP3 inflammasome activation. This attenuates oxidative stress, inflammatory responses, and neuronal degeneration, exerting a protective effect by inhibiting the NLRP3 inflammasome signaling pathway following subarachnoid hemorrhage (Zhang Z. H. et al., 2021). In summary, microglia mediate the regulatory effects of luteolin on neuroinflammation. By modulating the activation phenotype of microglia and suppressing inflammatory responses, luteolin not only alleviates central neuroinflammation but may also mitigate neuroinflammatory-related diseases through the interactive crosstalk between microglia and macrophages. Therefore, luteolin intervention targeting microglia represents a potential strategy for the treatment of neuroinflammatory disorders.

### 2.3 T cells

**FIGURE 2:**
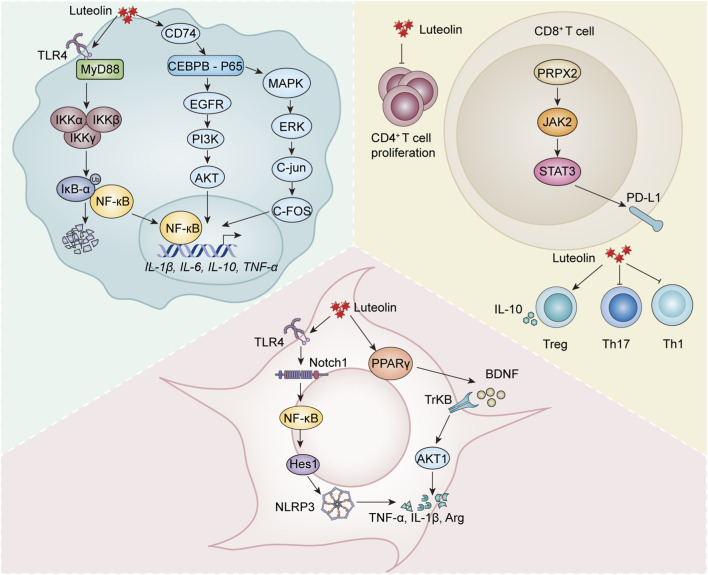
Molecular Mechanisms of Luteolin in Modulating Multicellular Immunity and Neuroprotection in Neurological Disorders. Luteolin exerts anti-inflammatory and neuroprotective effects on three key cell types: 1. Macrophages – inhibits TLR4/MyD88 and CD74/CEBPB-P65 signaling, blocks IKK-mediated IκBα degradation and NF-κB nuclear translocation, and suppresses PI3K/AKT and MAPK/ERK/AP-1 pathways, thereby reducing pro-inflammatory cytokines (IL-1β, IL-6, TNF-α) while maintaining IL-10.2. T cells – in CD8^+^ T cells, inhibits PRPX2/JAK2/STAT3 and downregulates PD-L1; in CD4^+^ T cells, promotes Treg differentiation with enhanced IL-10 and suppresses Th1/Th17 polarization, restoring immune homeostasis. 3. Microglia – activates PPARγ and BDNF/TrKB/AKT1 signaling to promote neuronal survival and synaptic plasticity, while inhibiting TLR4/NF-κB/Notch1/Hes1 and NLRP3 inflammasome assembly, reducing TNF-α, IL-1β, and arginase release.

T lymphocytes are central regulators of the adaptive immune response, playing indispensable roles in host defense against pathogens and malignancies. Upon antigen recognition through the TCR, naïve T cells undergo clonal expansion and differentiate into distinct effector subsets, including Th1, Th2, Th17, and Treg cells, each characterized by a unique cytokine profile and functional role (Mittelbrunn and Kroemer, 2021; Chung et al., 2024; Lin et al., 2024). The precise balance among these subsets is crucial for an effective and controlled immune response. Beyond its well-documented anti-inflammatory and antioxidant properties, luteolin exhibits significant immunomodulatory effects. Research has found that in lung adenocarcinoma, luteolin binds to PRDX2, inhibiting the JAK2/STAT3 pathway, reducing PD-L1 expression, and promoting the release of perforin and granzyme B by CD8^+^ T cells. This action counteracts immune evasion and suppresses the progression of lung adenocarcinoma (Li X. et al., 2025). In a rat liver transplantation model, luteolin modulates acute allograft rejection. It significantly protects the structure and function of the liver graft, prolongs the survival time of recipient rats, ameliorates T cell infiltration, and downregulates pro-inflammatory cytokines. Furthermore, it inhibits the proliferation of CD4^+^ T cells and Th cell differentiation while increasing the proportion of Tregs and suppressing Th1 differentiation, thereby providing immunosuppression for liver transplantation (Wang et al., 2023). In AR, luteolin reduces nasal symptom scores, elevates OVA-sIgG2a levels, and downregulates OVA-sIgE, OVA-sIgG1, and histamine levels in AR mice. It also increases Th1 and Treg cytokine levels while decreasing Th2 and Th17 cytokine levels, rectifying the imbalance in splenic T cell subsets. Additionally, it attenuates inflammatory cell infiltration and reduces the levels of ER stress-related and NLRP3 inflammasome activation-related mRNAs and proteins in the nasal mucosa, alleviating AR symptoms (Guo et al., 2025). Similarly, in an OVA-induced AR mouse model characterized by increased nasal sneezing frequency, nasal mucosal thickness, and elevated levels of anti-OVA-IgE, Beclin1, LC3II/LC3I, IL-17A, and RORγt, luteolin inhibits the expression of these factors, thereby restoring the Treg/Th17 balance and ameliorating AR (Yang et al., 2023). In hepatocellular carcinoma, luteolin enhances T cell activation, chemotaxis, and cytokine production. It helps maintain a high proportion of CD8^+^ T lymphocytes in the spleen, peripheral blood, and tumor tissue, augments the cytotoxicity of infiltrating CD8^+^ T lymphocytes, and promotes the generation of granzyme B, IFN-γ, and TNF-α in serum, thereby potentiating the anti-tumor effect (Cai et al., 2024). In esophageal cancer, combination therapy with luteolin and paclitaxel mitigates paclitaxel-induced hepatotoxicity, stimulates triggered release *in vivo*, and promotes specific cellular uptake and endosomal escape in esophageal cancer cells. This facilitates dendritic cell maturation and T cell infiltration, significantly activating the tumor immunosuppressive microenvironment and inhibiting esophageal cancer progression (Sun C. et al., 2025). In ARDS, luteolin significantly suppresses inflammation and alleviates CLP-induced lung injury *in vivo*. It inhibits the protein levels of caspase-11, caspase-1, GSDMD, IL-1α, and IL-1β in the lungs of model mice, promotes Treg frequency and IL-10 levels in the serum and BALF of CLP mice, and modulates Treg frequency as well as Treg-derived IL-10 levels, thereby ameliorating ARDS (Zhang Z. T. et al., 2021). In colon cancer, luteolin triggers ROS/Fe^2+^ accumulation, lipid peroxidation, elevated MDA, GSH depletion, and GPX4 downregulation. It directly binds to GPX4, enhancing its thermal stability and inhibiting proenzyme E-mediated degradation. This synergistically promotes intratumoral M1 macrophage polarization and CD8^+^ T lymphocyte activation, exerting anti-tumor effects (Cao et al., 2025). In conclusion, the regulation of neuroinflammation by luteolin is, in part, mediated through its effects on T cells. This compound can modulate the activation status and phenotypic differentiation of T cells, thereby suppressing inflammatory responses. While alleviating central neuroinflammation, it may also exert a holistic regulatory influence on neuroinflammatory-related diseases through the interplay between T cells and other immune cells. Therefore, T cell-targeted intervention strategies using luteolin hold promise as a novel potential avenue for the prevention and treatment of neuroinflammatory disorders (Figure 2).

## 3 Effects of luteolin on various neurological diseases

### 3.1 Alzheimer’s disease (AD)

The present review focuses on neurological disorders in which neuroinflammation plays a central pathogenic role and that are characterized by progressive neurodegeneration and/or motor dysfunction. AD is the most prevalent form of dementia worldwide, characterized by a progressive decline in cognitive function, memory impairment, and hallmark pathologies including extracellular Aβ plaques and intracellular neurofibrillary tangles composed of hyperphosphorylated tau protein (Ma et al., 2022; Kamatham et al., 2024; Lopez-Lee et al., 2024). Neuroinflammation in AD is primarily mediated by the innate immune cells of the CNS—microglia and astrocytes (Singh, 2022). This inflammatory milieu contributes to neuronal dysfunction and synaptic loss, and pro-inflammatory cytokines can drive further tau hyperphosphorylation and neurofibrillary tangle formation while also impairing glial cell function (Chen and Yu, 2023). Preclinical evidence from rodent models suggests that luteolin may improve disease specific outcomes. However, clinical evidence in humans is currently absent/very limited. As a naturally occurring potent modulator of neuroinflammation, luteolin exerts significant neuroprotective effects in neuroinflammatory-related disorders. Studies have demonstrated that in AD, luteolin treatment dose-dependently improves spatial learning and memory deficits in AD mouse models. This is accompanied by the inhibition of astrocyte overactivation (indicated by GFAP) and neuroinflammation (evidenced by reduced levels of TNF-α, IL-1β, IL-6, NO, COX-2, and iNOS protein) (Kou et al., 2022). Furthermore, luteolin decreases the expression of ER stress markers GRP78 and IRE1α in brain tissue. In rat glioma cells, luteolin treatment suppresses LPS-induced cell proliferation, excessive release of inflammatory cytokines, and the elevation of the ER stress marker GRP78, thereby contributing to cognitive improvement (Kou et al., 2022). Luteolin supplementation can also directly bind to and activate PPARγ, promoting its expression and function (He et al., 2023). This mechanism ameliorates memory and cognitive impairments in AD mice and exerts neuroprotective effects by inhibiting Aβ generation, repairing mitochondrial damage, and reducing neuronal apoptosis (He et al., 2023). Researchers have found that in the cortex and hippocampus of luteolin-treated AD mice, there is a reduction in Aβ-induced phosphorylation of JNK and p38 MAPK, alongside decreased levels of GFAP and Iba-1. Moreover, the expression of pro-apoptotic and anti-apoptotic markers such as Bax, Bcl-2, Caspase-3, and Cox-2 is significantly diminished, while the expression of synaptic markers like PSD-95 and SNAP-25 is markedly increased. Thus, luteolin may attenuate Aβ-related neuroinflammation and neurodegeneration, potentially through the inhibition of JNK signaling (Ahmad et al., 2021). ER stress is crucial for inflammation mediated via NF-κB and inflammasome activation. During the progression of AD, chronic inflammation associated with aging can prime microglia, leading to a hyper-responsive state upon stimulation. Luteolin treatment suppresses ER stress, reduces CD68 levels in the brain, and consequently inhibits LPS-induced IL-1β expression and depressive-like behaviors (Tana and Nakagawa, 2022). Combinatorial therapy with DHA, luteolin, and UA has shown enhanced efficacy in inhibiting Aβ pathology (Jayatunga et al., 2022). The development of novel nanoparticle formulations for luteolin delivery can also optimize its therapeutic effects. Compared to luteolin suspensions, these nanoparticles better improve short-term and long-term spatial memory acquisition, increase neuronal survival, reduce amyloid plaque burden, enhance antioxidant capacity, and suppress the release of pro-inflammatory factors, as well as inhibit Aβ aggregation and hyperphosphorylated tau protein levels, offering a more promising and safe nano-delivery system (Abbas et al., 2022; Elsheikh et al., 2022). Recent studies indicate that a co-ultramicronized composite of palmitoylethanolamide and luteolin (co-ultra PEALut) exerts superior therapeutic effects compared to luteolin alone, potentially by inhibiting astrocyte reactivity and inflammation while maintaining oligodendrocyte homeostasis, thereby improving memory function (Facchinetti et al., 2022). Therefore, luteolin demonstrates considerable potential in inhibiting AD progression and ameliorating cognitive function-related behavioral phenotypes.

### 3.2 Parkinson’s disease (PD)

PD is the second most common neurodegenerative disorder after AD. It is classically characterized by the progressive loss of dopaminergic neurons in the substantia nigra pars compacta and the presence of intraneuronal cytoplasmic inclusions known as Lewy bodies, which are primarily composed of misfolded α-synuclein (α-syn) (Bloem et al., 2021; Horsager and Borghammer, 2024; Iranzo et al., 2024). Accumulating evidence robustly indicates that neuroinflammation is a critical and persistent driver in the pathogenesis of PD (Araujo et al., 2022; Isik et al., 2023). It is not merely a secondary response to neuronal death but an active contributor to neurodegeneration. Microgliosis is widespread in brain regions affected by PD (Heidari et al., 2022; Kalyanaraman et al., 2024; She et al., 2025). As an effective natural modulator of neuroinflammation, luteolin plays a significant neuroprotective role in neuroinflammatory-related disorders. Research shows that luteolin treatment in rats downregulates the expression of components within the NF-κB/NLRP3 axis (including NF-κB, NLRP3, ASC, and Caspase-1), promotes increased levels of catalase and SOD in the hippocampus, and reduces protein levels of pro-inflammatory cytokines such as IL-6, IL-1β, and TNF-α. These effects ameliorate emotional disturbances in PD rats, exerting anxiolytic and antidepressant actions (She et al., 2025). Similarly, in a rotenone-induced Wistar rat model of PD, luteolin treatment elevates GSH levels and reduces nitrate content, restoring oxidative balance. It modulates the levels of TNF-α and Bax, thereby mitigating inflammation and neuronal apoptosis, promoting dopamine elevation, and improving PD pathology (Chib et al., 2025). In an SH-SY5Y cell PD model, luteolin exhibits no cytotoxicity. Its administration inhibits 6-OHDA-induced cell death by upregulating mRNA and protein expression of HRD1 and SEL1L, subsequently suppressing the ER stress response and exerting neuroprotection (Nishiguchi et al., 2024). In LPS-induced PC12 and BV2 cell models, luteolin treatment inhibits apoptosis and promotes PC12 cell survival. Furthermore, luteolin shifts microglial M1/M2 polarization towards the anti-inflammatory M2 phenotype both *in vitro* and *in vivo*. The underlying mechanism may involve inhibiting TLR4 transcription and translation, leading to suppressed phosphorylation of the NF-κB p65 subunit. This neuroprotective effect, including functional improvement and mitigation of dopaminergic neuron loss, has also been corroborated in rodent PD models (Xie et al., 2024). Consequently, luteolin demonstrates potential in slowing the progression of PD by modulating neuroinflammation-related pathways, suggesting its candidacy as a potential therapeutic compound for neuroinflammatory intervention in PD.

### 3.3 Multiple sclerosis (MS)

MS is a chronic, immune-mediated demyelinating disease of the CNS and a leading cause of non-traumatic neurological disability in young adults (Kuhlmann et al., 2023; Garton et al., 2024). Its pathogenesis involves multifocal inflammatory lesions, demyelination, axonal transection, and progressive neurodegeneration (Bar-Or and Li, 2021; Goris et al., 2022). In this condition, peripherally activated autoreactive T and B lymphocytes traverse the BBB and instigate a cascade of inflammatory responses within the CNS parenchyma. Following CNS infiltration, autoreactive CD4^+^ T cells, particularly those belonging to Th1 and Th17 lineages, become reactivated by local antigen-presenting cells (Jelcic et al., 2025; Laderach et al., 2025). This triggers a robust pro-inflammatory cytokine environment, including IFN-γ, IL-17, and GM-CSF, which further amplifies the immune response (Kumar et al., 2021; Martin et al., 2023). As an effective natural modulator of neuroinflammation, luteolin exerts important neuroprotective effects in neuroinflammatory-related diseases. In a mouse model of MS, motor coordination and spatial memory were assessed using the rotarod test and Morris water maze, respectively. Myelin integrity was evaluated via Luxol fast blue staining and MBP immunofluorescence/Western blot. Oxidative stress was measured by assessing SOD and GSH-Px activities, along with MDA levels. Nrf2 pathway activation was analyzed by examining Nrf2 nuclear translocation and the expression of downstream proteins (HO-1, NQO1) via Western blot. Molecular docking simulated the interaction between compounds and the Keap1 protein. Luteolin treatment promoted MBP expression, mitigated oxidative damage by modulating SOD, CAT, GSH-Px, and MDA levels, and preserved myelin integrity. It facilitated Nrf2 nuclear translocation and upregulated HO-1 and NQO1 expression, demonstrating stronger binding affinity to the target protein Keap1 (Tian et al., 2025). These actions restored motor coordination and spatial memory in mice, laying the foundation for developing luteolin-enriched dietary strategies (Tian et al., 2025). In a rat EAE model, compared to the EAE group, luteolin-treated EAE rats exhibited upregulated CNTF expression, significantly increased cAMP and TAC levels, and markedly decreased levels of cleaved caspase-3, NF-κB, and MIP-1α, indicating anti-inflammatory, anti-apoptotic, and neurotrophic effects (El-Deeb et al., 2019). The co-ultramicronized composite of palmitoylethanolamide and luteolin (co-ultra PEALut) promotes oligodendrocyte differentiation by increasing MBP, CNPase, and Tyro3 mRNA levels (Facci et al., 2021). By targeting neuroinflammatory pathways, luteolin exhibits potential in delaying the pathological progression of MS.

### 3.4 Stroke

Stroke is a leading global cause of death and long-term disability, encompassing two main pathological types: ischemic stroke, caused by occlusion of a cerebral artery, and hemorrhagic stroke, resulting from blood vessel rupture (Bushnell et al., 2024; Sharma and Lee, 2025). While the initial hypoxic-ischemic or mechanical insult determines the primary tissue damage, a rapidly evolving and complex neuroinflammatory cascade critically influences the extent of final brain injury, functional recovery, and neurological outcome (Marcucci et al., 2023; Seiffge et al., 2024). This secondary injury process involves a precisely timed interplay between resident glial cells and infiltrating peripheral immune cells, creating a dynamic inflammatory microenvironment that transitions from acutely detrimental effects to delayed repair mechanisms (Candelario-Jalil et al., 2022; Shi and Yong, 2025). Resident microglia rapidly transition to a reactive state, initially presenting a pro-inflammatory (M1-like) phenotype that releases an array of cytotoxic mediators, including cytokines (e.g., TNF-α, IL-1β), chemokines (e.g., CCL2, CXCL1), ROS, and MMPs (Kumari et al., 2024). These factors directly exacerbate neuronal death, disrupt the BBB, and promote the recruitment of peripheral leukocytes (Anthony et al., 2022). As an effective natural modulator of neuroinflammation, luteolin exerts significant neuroprotective effects in neuroinflammatory-related conditions. In OGD/R-treated BV2 cells and a cerebral I/R rat model, luteolin treatment significantly increased cell viability, reduced LDH release, and lowered intracellular ROS and MDA levels. It also enhanced the GSH/GSSG ratio and SOD activity in OGD/R-treated BV2 cells, markedly elevated protein levels of SLC7A11, NRF2, and GPX4, inhibited cell death, bolstered antioxidant function, suppressed ferroptosis, and reduced neurological scores and infarct volume in brain tissue of cerebral I/R rats (Li H. et al., 2025). Luteolin treatment can activate the SIRT3/AMPK/mTOR pathway, diminishing the number of GFAP- and Iba-1-positive glial cells in the hippocampus while enhancing the scavenging capacity of oxygen free radicals and SOD activity in mitochondria. By reversing mitochondrial swelling and restoring mitochondrial transmembrane potential, it augments antioxidant capacity and exerts neuroprotective effects following cerebral ischemia (Liu et al., 2020). In subarachnoid hemorrhage, luteolin treatment significantly enhanced the expression of Nrf2 while concurrently downregulating NLRP3 inflammasome activation. This attenuated oxidative stress, inflammatory responses, and neuronal degeneration, improved neurological function, and reduced neuronal cell death following subarachnoid hemorrhage (Zhang Z. H. et al., 2021). In a photothrombotic mouse model and an OGD cell model, luteolin alleviated cerebral infarction area and neuronal apoptosis, reduced brain water content and Evans blue leakage, decreased bEnd.3 cell apoptosis in the OGD model, improved transendothelial electrical resistance, and reduced FITC leakage. Luteolin also inhibited the degradation of tight junction proteins and decreased MMP9 expression, thereby ameliorating BBB permeability (Li et al., 2026). Accordingly, luteolin may provide neuroprotection following cerebral ischemia-reperfusion injury by inhibiting neuroinflammation and oxidative stress.

### 3.5 Depression

**FIGURE 3:**
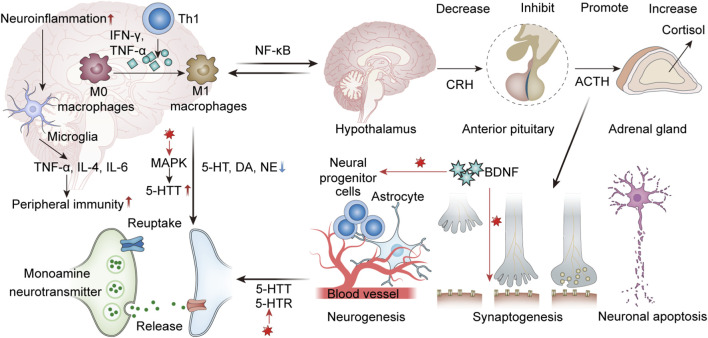
Multimodal Mechanisms of Luteolin in Regulating Neuroinflammation, Neuroendocrine Function, and Neuroplasticity in Neurological Disorders. Luteolin exerts neuroprotective effects by modulating four interconnected pathological axes: 1. Neuroinflammation and immunity – inhibits NF-κB and MAPK signaling, reducing pro-inflammatory cytokines (TNF-α, IL-6, IFN-γ) and suppressing M1 polarization of microglia/macrophages. 2. Monoamine homeostasis – downregulates 5-HTT, elevating extracellular 5-HT, DA, and NE to reverse neurotransmitter depletion. 3. HPA axis – reduces CRH and ACTH secretion, correcting cortisol overproduction and HPA axis hyperactivation. 4. Neuroplasticity & survival–upregulates BDNF, promotes neurogenesis and synaptogenesis, inhibits apoptosis, restoring neural circuit integrity.

Depression is a prevalent and debilitating psychiatric disorder characterized by persistent low mood, anhedonia, and cognitive impairments, imposing a substantial global disease burden (Pearce et al., 2022; Wang et al., 2022). For a long time, the pathophysiology of depression has been primarily explained through the framework of monoamine neurotransmitter dysregulation (Huang Y. Y. et al., 2024). Patients with major depressive disorder commonly exhibit elevated levels of pro-inflammatory cytokines in both peripheral and central compartments, such as IL-1β, IL-6, and TNF-α (Reyes-Martinez et al., 2023; Wu and Zhang, 2023). Furthermore, individuals with chronic inflammatory conditions or those receiving pro-inflammatory cytokine therapy show a significantly increased incidence of depressive symptoms (Ionescu et al., 2022). As an effective natural modulator of neuroinflammation, luteolin exerts important neuroprotective effects in neuroinflammatory-related disorders. In post-stroke depression, researchers, based on immune-related risk GO functions and bioinformatics algorithms, identified 335 immune-related risk GO functions and 37 compounds using PSD risk genes. Through the construction of a GO function network, STAT proteins were identified as potential pivot proteins in the PSD mechanism, and three key pathways—hsa04010 (MAPK signaling pathway), hsa04151 (PI3K-Akt signaling pathway), and hsa04060 (Cytokine-cytokine receptor interaction)—were explored, providing therapeutic strategies for post-stroke depression (Zhao et al., 2023). Under CUMS conditions, luteolin treatment upregulated Sirt1 expression and downregulated the expression of Ac-NF-κB, NLRP3, Ac-Caspase-1, GSDMD-N, cleaved IL-1β, and cleaved IL-18. It inhibited the secretion of pro-inflammatory factors IL-1β, IL-6, IL-18, and TNF-α in the hippocampus and corneal tissue, alleviated CUMS-induced depressive-like behaviors, increased tear secretion, and restored corneal defects in mice (Xie et al., 2023). In an AD mouse model, luteolin reduced CD68 levels in the brain, suppressed ER stress, inhibited LPS-induced IL-1β expression, and consequently curbed microglial activation in the brain, thereby enhancing locomotor activity and alleviating depressive-like behaviors (Tana and Nakagawa, 2022). Significant alterations in the glycerophospholipid metabolism pathway were observed in the hippocampus and prefrontal cortex of rats with late-onset depression. Luteolin treatment ameliorated depressive-like behaviors in these animals by specifically modulating glycerophospholipid metabolism in distinct brain regions and regulating autophagic dysfunction (Wu et al., 2024). In noise-induced depressive-like behavior, luteolin treatment significantly increased serum serotonin and norepinephrine levels in noise-exposed mice, ameliorated noise-induced inflammation in the hippocampus and prefrontal cortex, and increased synaptic protein levels, exerting antidepressant effects (Cheng et al., 2022). In rats subjected to chronic stress, characterized by monoamine imbalance and HPA axis overactivation, luteolin treatment reduced fear freezing responses during extinction recall, as well as depressive- and anxiety-like behaviors. It suppressed the elevation of plasma corticosterone and ACTH levels and restored the balance between increased norepinephrine and decreased serotonin levels in the fear circuit, medial prefrontal cortex, and hippocampus, exerting antidepressant and anxiolytic effects (Sur and Lee, 2022). In an LPS-induced neuroinflammation mouse model with concomitant astrocytic deficits, luteolin elevated mature BDNF, dopamine, and norepinephrine levels in the hypothalamus of LPS-induced depressive mice. It significantly reduced IL-6 production in mouse brain-derived astrocytes and serum, as well as serum TNFα and corticosterone levels, thereby attenuating the LPS-evoked inflammatory response, inhibiting neuroinflammation, and ameliorating depressive-like behaviors (Achour et al., 2021). In breast cancer-related depression, luteolin treatment significantly reduced tumor size and weight. It promoted the upregulation of miR-124–3p expression in the hippocampus, downregulated the expression of TNF-α and TRAF6, and decreased the phosphorylation levels of NF-κB and IκB. By modulating the miR-124-3p/TNF-α/TRAF6-related pathway, it inhibited neuroinflammation and improved breast cancer-related depression (Zhu et al., 2022). In summary, luteolin demonstrates potential therapeutic effects in intervening in the progression of depression by modulating neuroinflammation-related signaling pathways (Figure 3).

### 3.6 Epilepsy and seizures

Epilepsy is a common and serious neurological disorder characterized by an enduring predisposition to generate spontaneous, recurrent seizures (Kanner and Bicchi, 2022; Specchio et al., 2022). The pathogenesis of epilepsy is multifaceted and varies across different epilepsy syndromes. However, accumulating evidence points to neuroinflammation as a crucial mechanistic link between the initial brain insult, the occurrence of seizures, and the pathological remodeling of neural circuits that leads to chronic epilepsy (Ravizza et al., 2024; Hollis and Lukens, 2025). Once considered merely a consequence of seizures, neuroinflammation is now recognized as a dynamic and potent factor that can promote the initiation and perpetuation of hyperexcitable states (Suleymanova, 2021; Ramos et al., 2025). Seizures rapidly trigger the release of DAMPs and other signaling molecules from neurons, which engage pattern recognition receptors (such as Toll-like receptors, IL-1 receptors) on glial cells (AlAseeri et al., 2024; Hanin et al., 2024). This interaction triggers the production and release of a cascade of potent pro-inflammatory mediators, most notably including IL-1β, TNF-α, HMGB1, and complement proteins, which in turn modulate neuronal excitability (Fortunato et al., 2023; Vega Garcia et al., 2025). As an effective natural modulator of neuroinflammation, luteolin exerts significant neuroprotective effects in neuroinflammatory-related disorders. Research has shown that in a PTZ-induced rat model of epilepsy, luteolin reduces levels of cytokines TNF-α, IL-6, and IL-1β via the TLR4/IκBα/NF-κB pathway. This inhibition of the neuroinflammatory response mitigates hippocampal neuronal damage in PTZ-induced epileptic rats and partially restores behavioral function, as well as learning and memory abilities, thereby exerting neuroprotective effects and treating epilepsy (Cheng et al., 2024). In a kainic acid-induced mouse model of epilepsy, luteolin upregulates GADD45B, inhibits MAPK and NF-κB signaling pathways, and suppresses neuronal apoptosis and inflammation in kainic acid-induced epileptic mice, exerting neuroprotective and antiseizure effects (Luo et al., 2025). Notably, two independent studies using PTZ induced adult zebrafish reported no anticonvulsant effect of luteolin. Transcript levels of p70S6Kb, IL 1β, and caspase 3 remained unchanged 24 h post treatment, contrasting with rodent data. Potential explanations include: (i) species specific differences in luteolin metabolism; (ii) inadequate dosing relative to zebrafish physiology; (iii) different seizure induction protocols. This discrepancy underscores the need for cross species validation and standardized seizure models before concluding efficacy (Garbinato et al., 2021; Schneider et al., 2025). Based on these findings, luteolin may exert neuroprotective effects in epilepsy and seizure activity, potentially through the inhibition of neuroinflammatory responses.

### 3.7 Huntington’s disease (HD)

**FIGURE 4:**
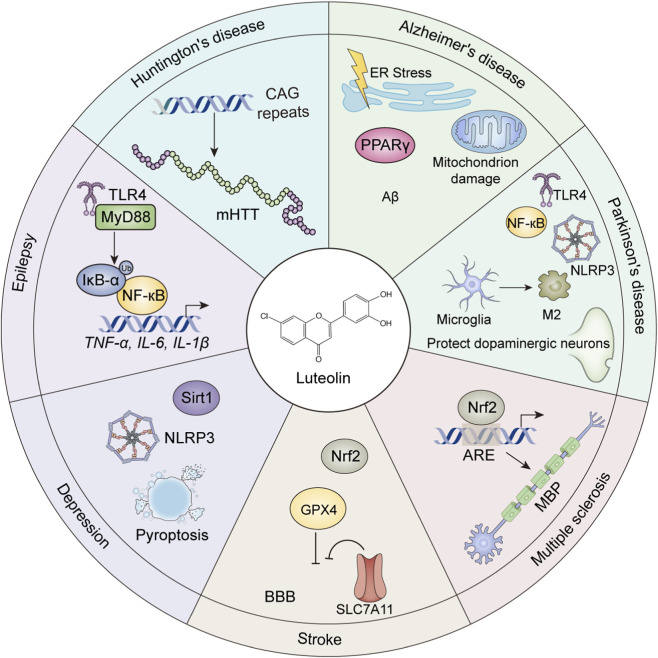
Multitarget Neuroprotective Effects of Luteolin in Major Neurological Disorders. Luteolin exerts disease-specific neuroprotective effects: 1. AD – activates PPARγ, reduces Aβ deposition, alleviates ER stress and mitochondrial damage. 2. PD – inhibits TLR4/NF-κB/NLRP3, promotes microglial M2 polarization, protects dopaminergic neurons. 3. MS– upregulates Nrf2/ARE, enhances MBP expression, supports oligodendrocyte function for myelin repair. 4. Stroke– activates Nrf2/GPX4 to reduce oxidative stress, maintains BBB integrity via SLC7A11.5. Depression – inhibits NLRP3 inflammasome and pyroptosis, reducing neuroinflammation. 6. Epilepsy – suppresses TLR4/MyD88/NF-κB, decreases pro-inflammatory cytokines (TNF-α, IL-6, IL-1β). 7. HD–targets mutant huntingtin aggregates, reducing proteotoxic stress and neuronal dysfunction.

HD is a devastating autosomal dominant neurodegenerative disorder caused by an abnormal expansion of CAG trinucleotide repeats within the HTT gene, leading to the production of mutant huntingtin protein (mHTT) with a toxic polyglutamine tract (Tabrizi et al., 2022a; Tabrizi et al., 2022b; Tong et al., 2024). Although HD is typically defined by the selective and progressive degeneration of medium spiny neurons in the striatum, resulting in severe motor, cognitive, and psychiatric symptoms, mutant huntingtin is expressed not only in neurons but also in microglia and astrocytes, where it directly disrupts their homeostatic functions (Brandebura et al., 2023; Abedrabbo et al., 2025). In microglia, mHTT impairs mitochondrial dynamics, promotes a chronically reactive state, and alters transcriptional responses to stimuli, leading to excessive and sustained production of pro-inflammatory cytokines such as IL-1β, IL-6, and TNF-α (Field et al., 2024; Sadeghdoust et al., 2024). As an effective natural modulator of neuroinflammation, luteolin exerts significant neuroprotective effects in neuroinflammatory-related disorders. In N171-82Q transgenic HD mice and wild-type littermates treated with luteolin starting at 6 weeks of age, serum NfL levels were reduced, and the accumulation of huntingtin aggregates in the cerebral cortex, hippocampus, and striatum was significantly diminished. This treatment also improved survival rates and prevented weight loss in HD mice (Mohammed et al., 2024). Luteolin reduced soluble and insoluble mutant huntingtin aggregation in HD mutant neuroblastoma cells, increased cell viability, and decreased apoptosis, protecting cultured cells from the cytotoxic effects of mutant huntingtin. This provides a novel potential therapeutic and protective strategy for mitigating cytotoxicity in neurodegenerative diseases like HD (Ramadan et al., 2023). In summary, luteolin demonstrates potential therapeutic value in delaying the pathological progression of HD by modulating molecular pathways associated with neuroinflammation (Figure 4).

### 3.8 Migraine and nervous system tumors

Migraine affects nearly one billion people worldwide and represents a serious neurological disorder whose pathogenesis is largely driven by neuroinflammation, involving the activation of trigeminal afferents and the release of calcitonin gene-related peptide (CGRP) and substance P (Peters, 2019; Pensato et al., 2023). Concurrently, primary brain tumors, particularly glioblastoma (GBM), are characterized by a highly inflammatory tumor microenvironment in which glioma-associated microglia and macrophages (GAMs) promote tumor progression, immune evasion, and therapy resistance (Hamad et al., 2023; Schaff and Mellinghoff, 2023; Salvalaggio et al., 2024). Accumulating evidence suggests that chronic neuroinflammation may serve as a shared mechanistic link between these two disease entities. Luteolin, a natural flavonoid, has been reported to exert key therapeutic potential against migraine by alleviating neuroinflammation and efficiently binding to CGRP protein with a binding affinity of −7.63 kcal/mol (PDB ID: 6PFO); its primary targets include PTGS2, AKT1, ESR1, MMP2, and MMP9. Although luteolin is well absorbed orally, its clinical translation is constrained by low water solubility, limited skin permeability, and blood-brain barrier (BBB) restriction, necessitating nanoformulation strategies to enhance intracerebral delivery and requiring *in vivo* validation of efficacy (Rushendran and Vellapandian, 2024). As one of the active compounds in Toutongning Capsule (TTNC), luteolin exerts a significant vasodilatory effect by relaxing cerebrovascular smooth muscle cell spasm, thereby primarily alleviating non-neuronal dysfunction (e.g., aberrant vascular smooth muscle contraction) in migraine (Zhang et al., 2024). Studies have further demonstrated that luteolin dually inhibits the malignant progression of glioma stem cells (GSCs) and improves the immune microenvironment by suppressing GSC-mediated IL6/STAT3 and TGFβ1/SMAD3 signaling pathways while modulating the polarization of tumor-associated macrophages toward the M2 phenotype, highlighting its potential as an anti-glioma agent and a targeted microenvironment therapy (Zong et al., 2024). In glioblastoma, luteolin concurrently induces apoptosis and protective autophagy; combination with an autophagy inhibitor enhances its anti-tumor efficacy, offering a potential strategy to improve GBM prognosis (Lee H. S. et al., 2021). Natural flavonoid-coordinated Fe^3+^ nanoparticles loaded with dihydroartemisinin (DHA) upregulate heme oxygenase-1 (HO-1), deplete glutathione, mediate photothermal effects, and modulate tight junction proteins of the BBB, synergistically inducing quadruple-amplified ferroptosis and achieving potent anti-GBM activity (Liu et al., 2025). In GBM stem cells, luteolin effectively suppresses tumor cell survival and sensitizes cells to temozolomide chemotherapy by modulating sphingolipid homeostasis (inhibiting SphK1/2, upregulating SGPL1 and CERS1), blocking pro-tumor signaling pathways such as MAPK and PI3K/AKT/mTOR, activating apoptotic mediators, and inhibiting protective autophagy (Navone et al., 2023). Collectively, luteolin may exert neuroprotective effects during the onset of migraine and nervous system tumors by suppressing neuroinflammatory responses.

## 4 Conclusion and future perspectives

**FIGURE 5:**
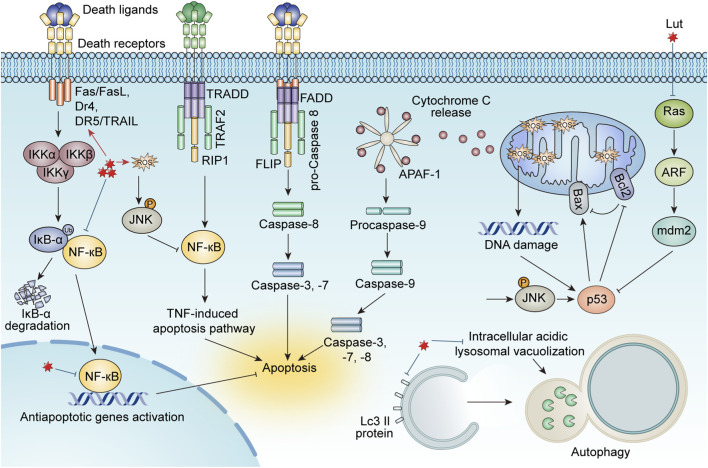
Mechanisms of Luteolin in Regulating Cell Death Programs (Apoptosis and Autophagy) and Stress Response Signaling Pathways in Neurological Disorders. Luteolin exerts neuroprotective effects by targeting core pathways of neuronal death and stress responses: 1. Inhibition of death receptor-mediated apoptosis: Luteolin blocks the activation of death receptor complexes (Fas/FasL, Dr4/DR5/TRAIL), inhibits IKKα/β/γ complex-mediated IκB-α degradation, thereby preventing NF-κB nuclear translocation and pro-apoptotic signaling; it also suppresses JNK activation, attenuating TNF-induced apoptotic pathway activation. 2. Inhibition of the mitochondrial apoptotic pathway: Luteolin reduces ROS-induced mitochondrial dysfunction, blocks cytochrome c release, regulates the Bcl-2/Bax balance to inhibit mitochondrial outer membrane permeabilization, and subsequently suppresses caspase-9 activation and the downstream effector caspase (caspase-3, -7, -8) cascade, thereby blocking mitochondria-dependent apoptosis. 3. Regulation of p53-mediated DNA damage response: Luteolin inhibits the Ras/ARF/mdm2 signaling pathway, reduces p53 phosphorylation and activation, decreases p53-mediated transcription of pro-apoptotic genes, and alleviates DNA damage-induced neuronal apoptosis. 4. Induction of neuroprotective autophagy: Luteolin promotes autophagosome formation (upregulation of Lc3-II protein) and accelerates lysosomal degradation of cytotoxic aggregates and damaged organelles, inducing adaptive autophagy to clear cytotoxic materials and reduce neuronal stress and death.

In summary, the substantial body of evidence reviewed herein clearly indicates that luteolin, as a multi-target natural flavonoid compound, exhibits significant neuroprotective potential across a diverse range of disease models associated with neuroinflammation (Li M. et al., 2025; Sun P. et al., 2025; Wan et al., 2025). Its mechanisms of action are multifaceted and multidimensional. Firstly, luteolin can directly intervene in key pathological processes, such as inhibiting Aβ aggregation and tau hyperphosphorylation in AD models, and mitigating oxidative stress-induced dopaminergic neuron damage in PD models (Kou et al., 2022; Tao et al., 2023; She et al., 2025). Secondly, it exerts anti-inflammatory effects by modulating several core signaling pathways, including the inhibition of NF-κB and MAPK pathway activation, as well as interfering with NLRP3 inflammasome assembly (Lee M. N. et al., 2021; Huang R. et al., 2024). This leads to a marked reduction in the production of pro-inflammatory cytokines such as IL-1β, TNF-α, and IL-6, effectively curtailing the neuroinflammatory cascade (Huang R. et al., 2024; Luo et al., 2026; Ouyang et al., 2026). Furthermore, luteolin activates the Nrf2/ARE signaling pathway, enhancing the activity of antioxidant enzymes like SOD and GSH-Px, while reducing MDA levels, thereby alleviating oxidative stress injury (Cao et al., 2025; Ding et al., 2025; Xiang et al., 2025). In terms of anti-apoptosis, it inhibits the mitochondria-mediated apoptotic cascade by modulating the expression ratio of Bcl-2 family proteins (Rath et al., 2024; Zhai et al., 2024). Notably, its ability to reduce infarct volume and improve neurological deficits in cerebral ischemia models, as well as to modulate monoamine neurotransmitter levels in depression models, provides further functional evidence supporting its multifaceted therapeutic value (Li H. et al., 2025; Li et al., 2026). Shared mechanisms across all diseases include inhibition of NF-κB and NLRP3 inflammasomes, activation of Nrf2, and suppression of pro-inflammatory cytokines. Disease-specific mechanisms include: Aβ aggregation inhibition in AD, α-synuclein reduction in PD, myelin repair in MS, ferroptosis inhibition in stroke, monoamine modulation in depression, seizure suppression via GADD45 B in epilepsy, and mHTT aggregation reduction in HD. Luteolin exhibits low oral bioavailability (0.5%–2% in rats, estimated <1% in humans) due to extensive phase II metabolism (glucuronidation/sulfation) in the gut and liver. Peak plasma concentration (Cmax) after oral administration is typically <100 ng/mL, far below effective *in vitro* concentrations (1–50 µM). Interspecies scaling suggests a human equivalent dose of 5–10 mg/kg/day, but this remains unvalidated (Boersma et al., 2002; Miyashita et al., 2022). Most preclinical studies on luteolin lack blinding, randomization, and sample size justification, and almost all used only male animals with short treatment durations. Negative findings are rarely reported, suggesting publication bias. Future investigations must adopt rigorous experimental designs (e.g., ARRIVE guidelines) to improve reproducibility and translational validity (Figure 5).

Despite these encouraging preclinical findings, the translation of luteolin into a clinically viable therapeutic agent faces several challenges. Current research is predominantly confined to cellular and animal models; its neuroprotective efficacy, pharmacokinetic profile, and long-term safety within the complex physiological environment of the human body remain to be fully elucidated. Concurrently, its precise interaction network with multiple molecular targets requires systematic investigation and validation through advanced pharmacological studies. A critical limiting factor is the poor water solubility and low oral bioavailability of luteolin, which poses a substantial obstacle to its further pharmaceutical development. Therefore, future research endeavors should concentrate on the following core directions: precisely mapping its molecular target landscape utilizing chemical biology, network pharmacology, and gene editing technologies; developing innovative nano-delivery systems or structural modification strategies to enhance its *in vivo* distribution, particularly its bioavailability and ability to cross the BBB; and ultimately, conducting rigorously designed clinical trials to confirm its safety, tolerability, and efficacy in patients with neuroinflammation-related disorders. Overcoming these translational bottlenecks is paramount to advancing luteolin from a promising preclinical candidate to a transformative therapeutic agent for the intervention of neurodegenerative and other neuroinflammation-associated diseases.
